# Systemic Therapies for Moderate-to-Severe Atopic Dermatitis in Children and Adolescents: A Systematic Review

**DOI:** 10.7759/cureus.94907

**Published:** 2025-10-19

**Authors:** Sahar Khalil Hamza Osman, Malaz Awad Mohamed Ahmed, Hoda Idrees, Aziza Mohammad Hassan Mohammad Ali, Aisha Hassan Ahmed Taha, Fatima Hassan Musa Shaikhelsafi, Afrah Mohamed Mirghani Hamour

**Affiliations:** 1 Internal Medicine, Warwick Hospital, Coventry, GBR; 2 Internal Medicine, The Shrewsbury and Telford Hospital NHS Trust, Shrewsbury, GBR; 3 Faculty of Medicine, University of Medical Sciences and Technology (UMST), Khartoum, SDN; 4 General Practice, NHS England - West Midlands, Birmingham, GBR; 5 Dermatology, Dr. Health Clinics, Riyadh, SAU; 6 Dermatology, North West Armed Forces Hospital, Ministry of Defense Health Services, Tabuk, SAU; 7 Dermatology and Venereology, Qassim Specialist Medical Center, Ar Rass, SAU; 8 Dermatology, Najran Armed Forces Hospital, Ministry of Defense Health Services, Najran, SAU

**Keywords:** abrocitinib, adolescent, atopic dermatitis, biologic therapy, child, dupilumab, eczema, janus kinase inhibitors, systematic review, upadacitinib

## Abstract

Moderate-to-severe atopic dermatitis (AD) in children and adolescents imposes a significant burden, often requiring systemic therapy. With the recent development of targeted biologics and Janus kinase (JAK) inhibitors, the treatment landscape has evolved rapidly. This systematic review aims to critically evaluate the efficacy and safety of these advanced systemic therapies in the pediatric population. A systematic search of PubMed/MEDLINE, Scopus, Web of Science, and ClinicalTrials.gov was conducted for randomized controlled trials (RCTs) published from 2020 onwards, yielding 250 records. Thirteen studies involving over 3,500 pediatric patients were included. Studies evaluating biologics or JAK inhibitors in children and adolescents (0-18 years) with moderate-to-severe AD were included. The Cochrane Risk of Bias 2 (ROB 2) tool was used for quality assessment. A narrative synthesis was performed due to clinical heterogeneity. Dupilumab and tralokinumab (biologics) demonstrated significant efficacy, with Eczema Area and Severity Index 75 (EASI-75) response rates of 43.3% at week 16 and sustained improvements in disease severity (SCORAD, IGA) and pruritus. The JAK inhibitors, abrocitinib and upadacitinib, showed rapid and high-magnitude efficacy, with EASI-75 and Validated Investigator Global Assessment (vIGA-AD) response rates frequently exceeding 70-90% by weeks 12-16 and providing rapid itch relief. Dupilumab’s safety profile was favorable, with mostly mild-to-moderate adverse events (e.g., conjunctivitis). JAK inhibitors were associated with acne, nausea (abrocitinib), and herpes infections, necessitating routine monitoring. The overall risk of bias was low across most studies. Advanced systemic therapies are highly effective for moderate-to-severe pediatric AD. Biologics offer a well-established safety profile, while JAK inhibitors provide superior and faster efficacy, particularly for itch, but require careful safety monitoring. Treatment choice should be individualized based on disease severity, preference, and risk profile.

## Introduction and background

Atopic dermatitis (AD) is a chronic, relapsing inflammatory skin disease that affects a substantial proportion of children and adolescents worldwide [1]. Characterized by pruritus, eczematous lesions, and recurrent flares, AD imposes a considerable physical, psychological, and social burden on affected patients and their families [2]. Global prevalence estimates suggest that up to 20% of children experience AD at some point during childhood, with a smaller but significant proportion developing moderate-to-severe forms of the disease. These more severe phenotypes are frequently associated with sleep disturbance, impaired quality of life, comorbid allergic conditions, and increased healthcare utilization [3].

First-line therapies, including emollients, topical corticosteroids, and calcineurin inhibitors, remain the cornerstone of management for mild-to-moderate disease [4]. However, in children and adolescents with moderate-to-severe AD, topical treatment alone is often insufficient to achieve long-term disease control. For these patients, systemic therapies may be required to reduce disease activity, minimize flares, and improve quality of life [5]. Conventional systemic immunosuppressants, such as cyclosporine, methotrexate, azathioprine, and mycophenolate mofetil have been used in pediatric populations, though their long-term safety and efficacy remain uncertain [6]. More recently, advances in understanding the immunopathogenesis of AD have led to the development of targeted biologic agents and small molecules, including dupilumab, tralokinumab, and Janus kinase (JAK) inhibitors, which show promising results in adults and are increasingly being studied in younger populations [7].

Despite these emerging therapeutic options, evidence guiding the optimal use of systemic agents in children and adolescents with moderate-to-severe AD is limited [8]. Clinical decision-making is often challenged by the scarcity of high-quality comparative studies, heterogeneity in outcome measures, and concerns regarding long-term safety in this vulnerable age group [9]. A systematic evaluation of the available literature is therefore essential to synthesize current evidence, identify knowledge gaps, and inform clinical practice.

This systematic review aims to critically evaluate the efficacy and safety of systemic therapies for moderate-to-severe AD in children and adolescents. By consolidating evidence from randomized controlled trials (RCTs), this review seeks to provide clinicians and policymakers with an updated and comprehensive overview of therapeutic options available for this population.

## Review

Methodology

This systematic review was conducted and reported in accordance with the Preferred Reporting Items for Systematic Reviews and Meta-Analyses (PRISMA) 2020 statement to ensure a comprehensive and transparent process [10].

Eligibility Criteria

Studies were selected based on predefined eligibility criteria to ensure the inclusion of relevant and high-quality evidence. The population included children (0-12 years) and adolescents (13-18 years) with a physician-confirmed diagnosis of moderate-to-severe AD inadequately controlled by topical therapies. Disease severity was defined according to validated clinical criteria, such as an Eczema Area and Severity Index (EASI) score ≥16, a Scoring Atopic Dermatitis (SCORAD) score ≥25, or an Investigator’s Global Assessment (IGA) score ≥3. Interventions included all systemic pharmacological therapies, such as biologics (e.g., dupilumab, tralokinumab) and JAK inhibitors (e.g., upadacitinib, abrocitinib), including studies reporting different dosing regimens by age group (e.g., weight-based doses for children and fixed doses for adolescents). Comparators included placebo, another active systemic therapy, or different dosing regimens of the same intervention. The main outcomes were efficacy, measured by validated scales such as EASI-75 and IGA 0/1, and safety, assessed through the incidence of adverse and serious adverse events. Both short-term (≤16 weeks) and long-term (>16 to 52 weeks) observations were considered to evaluate early and sustained effects. Only RCTs published in English between January 1, 2020, and the search date in 2025 were included to ensure the review reflects the most recent and reliable evidence.

Information Sources and Search Strategy

A systematic and comprehensive search strategy was developed to identify all relevant published and unpublished studies. The following electronic bibliographic databases were searched from their inception to the present date: PubMed/MEDLINE, Scopus, and Web of Science. Furthermore, the ClinicalTrials.gov registry was searched to identify any completed but unpublished trials or ongoing studies to mitigate publication bias. The search strategy was developed in consultation with a medical librarian and utilized a combination of Medical Subject Headings (MeSH) terms and free-text keywords related to the core concepts: "atopic dermatitis", "eczema", "child", "adolescent", "pediatric", and the names of all known systemic therapies. The search strategy was first developed for PubMed/MEDLINE and subsequently adapted for the syntax and subject headings of the other databases.

Study Selection Process

The results from all database searches were imported into the Covidence systematic review software, where duplicate records were automatically and manually identified and removed. The study selection process was conducted in two phases by two independent reviewers (SKHA and HI). In the first phase, reviewers screened the titles and abstracts of all retrieved records against the eligibility criteria. In the second phase, the full texts of all potentially relevant studies were obtained and assessed in detail for final inclusion. Any disagreements between the reviewers at either stage were resolved through discussion or, if necessary, by consultation with a third senior reviewer (AMMH). The study selection process was documented using a Preferred Reporting Items for Systematic Reviews and Meta-Analyses (PRISMA) flow diagram, which detailed the number of records identified, included, and excluded at each stage, along with the specific reasons for exclusion at the full-text stage.

Data Collection Process and Data Items

A standardized data extraction form was pilot-tested on two included studies and then used to extract data from all eligible studies. Data extraction was performed independently by two reviewers (SKHA and HI) to ensure accuracy and consistency. Any discrepancies in the extracted data were resolved by consensus. The following data were extracted from each included study: (1) study characteristics (first author, publication year, country, funding source, trial registration number); (2) participant characteristics (sample size, age range, mean age, baseline disease severity, prior treatments); (3) intervention and comparator details (drug name, dosage, frequency, duration of treatment); and (4) outcome data for all pre-specified efficacy and safety endpoints, including means, standard deviations, and proportions for each study arm at relevant time points.

Risk of Bias Assessment

The methodological quality and risk of bias of each included RCT were critically appraised using the Cochrane Risk of Bias tool for randomized trials (ROB 2) [11]. This tool assesses bias across five domains: (1) bias arising from the randomization process, (2) bias due to deviations from intended interventions, (3) bias due to missing outcome data, (4) bias in measurement of the outcome, and (5) bias in selection of the reported result. Two reviewers (SKHA and HI) independently assessed each study and assigned a judgment of "low risk of bias," "some concerns," or "high risk of bias" for each domain. An overall risk of bias judgment for each study was then determined based on the domain-level judgments. Disagreements were resolved through discussion. The results of the risk of bias assessment are presented in a table and summarized in a narrative synthesis.

Synthesis Methods

Although only RCTs were included, substantial clinical and methodological heterogeneity was observed across studies, including differences in intervention types (injectable biologics vs. oral JAK inhibitors), dosing regimens, treatment durations, patient age ranges, and outcome measures. While we considered conducting a meta-analysis for common interventions, the limited number of directly comparable RCTs for each specific drug and dosing regimen made quantitative synthesis and reliable heterogeneity assessment (e.g., I² statistics, subgroup or sensitivity analysis, and meta-regression) impractical. Applying such statistical methods to highly variable data could lead to misleading conclusions. Therefore, a narrative synthesis was performed, organizing findings by intervention type and outcomes, and summarizing the direction, magnitude, and consistency of effects in both tables and text to provide an accurate and context-based interpretation of the evidence.

Results

Study Selection Process

**Figure 1:**
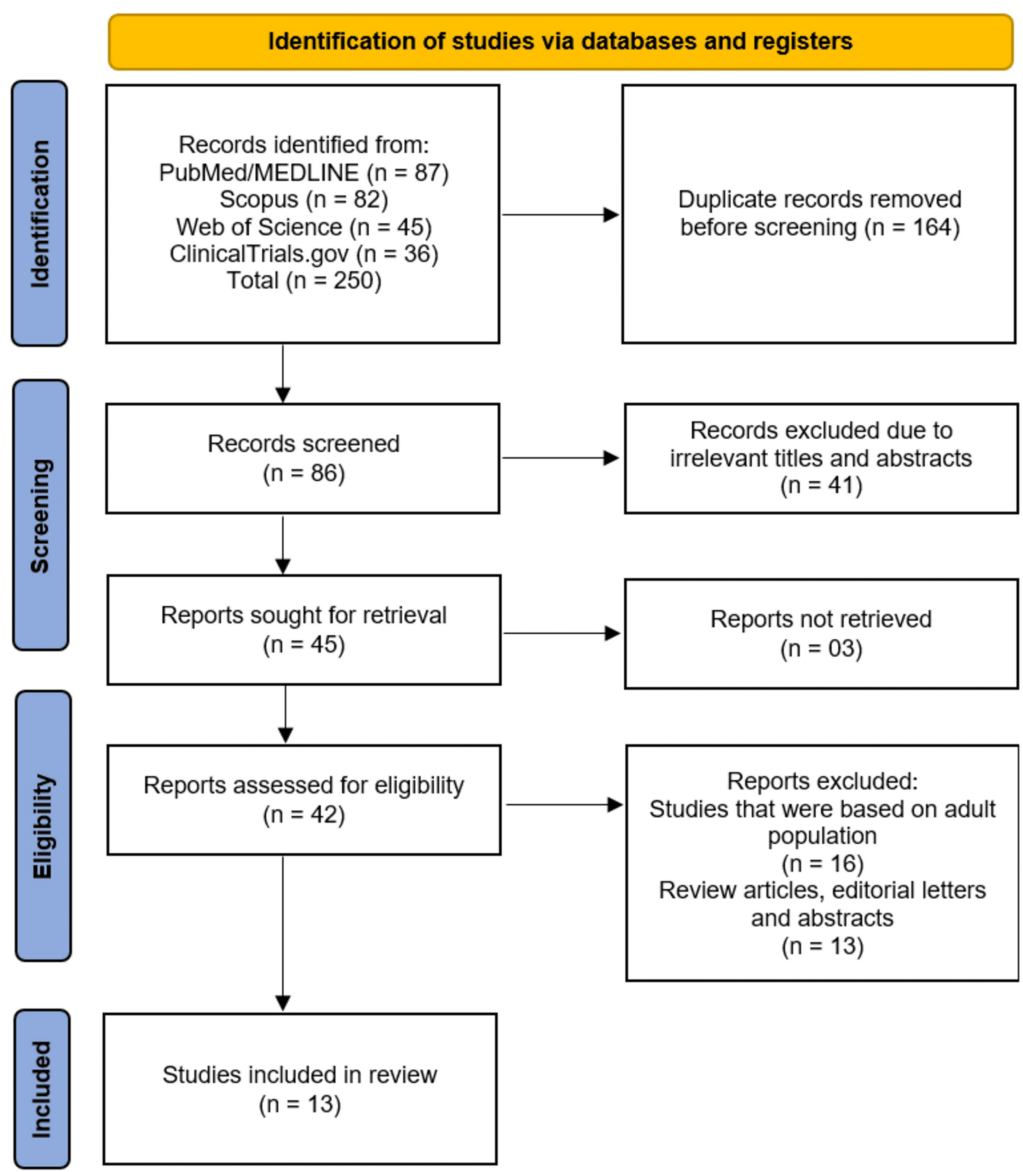
Illustration of the Studies' Selection Process on the PRISMA Flowchart PRISMA: Preferred Reporting Items for Systematic Reviews and Meta-Analyses

The study selection process is detailed in the PRISMA flow diagram (Figure 1). A systematic search of electronic databases (PubMed/MEDLINE, Scopus, Web of Science) and the clinical trial registry ClinicalTrials.gov yielded a total of 250 records. After the removal of 164 duplicate records, 86 unique records were screened based on their titles and abstracts. This initial screening phase led to the exclusion of 41 records that were deemed irrelevant to the review's focus. The full texts of the remaining 45 reports were sought for retrieval, of which three could not be obtained. Consequently, 42 full-text articles were assessed for eligibility. Of these, 29 were excluded for the following reasons: 16 studies were based solely on an adult population, and 13 were review articles, editorial letters, or conference abstracts that did not present new primary data. This process resulted in the inclusion of 13 studies [12-24] that met all pre-defined eligibility criteria for this systematic review.

Study Characteristics

A total of 13 studies [12-24] were included in this systematic review, comprising RCTs and post-hoc analyses of RCTs. The key characteristics of these studies are summarized in Table 1. The studies were published between 2020 and 2025, reflecting the recent advancements in systemic therapies for pediatric AD. The sample sizes varied, with several large multinational trials [12,15,16,19,20,24] and others conducted specifically in Japan [13,17,18]. The enrolled populations spanned different pediatric age groups: young children (six months to five years) [14], children (6-<12 years) [24], and adolescents (12-17 years) [12,15,16,19-23]. Most studies were phase 3, double-blind, placebo-controlled trials with primary endpoints assessed at 12 or 16 weeks, though several included long-term extension phases with follow-up durations extending to 52, 76, and 112 weeks, and beyond [12,15-17,24].

**Table 1:** Key Characteristics of the Included Studies AD: Atopic Dermatitis; CDLQI: Children’s Dermatology Life Quality Index; EASI: Eczema Area and Severity Index; EASI-50/75/90: 50%/75%/90% improvement from baseline in EASI; ECZTRA: Eczema Tralokinumab Clinical Trial Program; IGA: Investigator’s Global Assessment; NRS: Numeric Rating Scale; WSI-NRS: Worst Scratch/Itch Numeric Rating Scale; WP-NRS: Worst Pruritus Numeric Rating Scale; PP-NRS4: Peak Pruritus Numeric Rating Scale (≥4-point improvement); RCT: Randomized Controlled Trial; SCORAD: Scoring Atopic Dermatitis; SCORAD-50: 50% reduction in SCORAD; SD: Standard Deviation; TEAE: Treatment-Emergent Adverse Event; TCS: Topical Corticosteroids; vIGA-AD: Validated Investigator’s Global Assessment for Atopic Dermatitis; OLE: Open-Label Extension

Author (Year)	Country	Study Design	Sample Size (Children/Adolescents)	Age Range	Intervention (Drug, Dose, Duration)	Comparator	Follow-up Duration	Primary Outcome(s)
Paller et al., [12] 2025	Multinational (ECZTRA 6, clinical trial sites across multiple countries)	Phase 3 RCT, Post hoc analysis	247 adolescents	Not specified (adolescent population, typically 12–17 years)	Tralokinumab 150 mg or 300 mg, subcutaneous, 16 weeks (with open-label extension up to 52 weeks)	Placebo	16 weeks (primary); 52 weeks (extension)	EASI-50, ≥3-point improvement in itch NRS, ≥6-point improvement in CDLQI, IGA 0/1
Ebisawa et al., [13] (2024)	Japan	Randomized, double-blind, phase 3 RCT	62 (Dupilumab: 30; Placebo: 32)	≥6 months to <18 years	Dupilumab + topical corticosteroids, 16 weeks (then open-label dupilumab to 52 weeks)	Placebo + topical corticosteroids	52 weeks (primary endpoint at 16 weeks)	Proportion achieving EASI-75 at Week 16
Boguniewicz et al., [14] (2024)	Multicenter, international – LIBERTY AD PRESCHOOL trial	Randomized, placebo-controlled trial (post hoc analysis)	162	6 months – 5 years	Dupilumab (dose and regimen not specified in abstract), 16 weeks	Placebo	16 weeks	Investigator’s Global Assessment (IGA 0/1), EASI-75, ≥4-point reduction in Worst Scratch/Itch Numeric Rating Scale (WSI-NRS)
Paller et al., [15] (2025)	Multicenter	Phase 2/3 RCTs + Extension Study	170 (200 mg), 187 (100 mg) for efficacy; 289 (200 mg), 201 (100 mg) for safety	Adolescents (exact age range not specified)	Abrocitinib 200 mg or 100 mg orally, daily, median exposure: 971 days (200 mg), 899 days (100 mg)	Placebo or other trial-specific comparators (not detailed in abstract)	Up to 112 weeks for efficacy; up to 4.6 years for safety	Proportion achieving IGA 0/1, EASI-75, EASI-90; incidence of treatment-emergent adverse events (TEAEs) and serious TEAEs
Paller et al., [16] (2024)	Multinational	Phase 3, double-blind, placebo-controlled RCTs	542 adolescents	12–17 years	Upadacitinib 15 mg or 30 mg, oral, once daily; duration up to 76 weeks	Placebo (with or without topical corticosteroids)	76 weeks	EASI-75, vIGA-AD score 0/1 with ≥2-grade improvement, WP-NRS improvement ≥4 points
Katoh et al., [17] (2023)	Japan	Phase 3, Randomized, Multicenter	272 (UPA15: 120; UPA30: 122)	Not specified (adolescents included)	Upadacitinib 15 mg or 30 mg + topical corticosteroids, 112 weeks	Placebo (rerandomized to UPA15 or UPA30 at week 16)	112 weeks (2-year interim)	EASI 75/90, vIGA-AD 0/1, WP-NRS ≥4-point improvement
Katoh et al., [18] (2022)	Japan	Phase 3, double-blind, randomized	272 (children/adolescents not separately specified)	12–75 years	Upadacitinib 15 mg + TCS or 30 mg + TCS	Placebo + TCS	24 weeks	Safety (adverse events, laboratory data)
Reich et al., [19] (2021)	22 countries (Asia-Pacific, Europe, Middle East, North America, Oceania)	Randomized, double-blind, placebo-controlled, phase 3 trial	Adolescents: 12–17 years (subset of 901 total patients; exact adolescent numbers not specified)	12–17 years	Upadacitinib 15 mg or 30 mg + topical corticosteroids, once daily for 16 weeks	Placebo + topical corticosteroids	16 weeks	EASI-75 at week 16; vIGA-AD response at week 16
Guttman-Yassky et al., [20] (2021)	24 countries (Measure Up 1) and 23 countries (Measure Up 2) across Europe, North & South America, Oceania, Asia-Pacific	Multicentre, Randomized, Double-blind, Placebo-controlled, Phase 3	Measure Up 1: 281 (15 mg) + 285 (30 mg) + 281 (placebo); Measure Up 2: 276 (15 mg) + 282 (30 mg) + 278 (placebo) – adolescents included in overall population	12–17 years (adolescents; adults also included)	Upadacitinib 15 mg or 30 mg orally, once daily, 16 weeks	Placebo	16 weeks	Coprimary: EASI-75 (≥75% improvement from baseline) and vIGA-AD response (score 0/1 with ≥2-grade reduction) at week 16
Eichenfield et al., [21] (2021)	Asia–Pacific, Europe, North America	Phase 3, randomized, double-blind, placebo-controlled	285 adolescents (145 boys, 140 girls)	12–17 years	Oral abrocitinib 200 mg or 100 mg once daily + topical therapy for 12 weeks	Placebo + topical therapy	12 weeks	Coprimary: IGA 0/1 response (clear/almost clear with ≥2-grade improvement); EASI-75 response at week 12; Key secondary: PP-NRS4 (≥4-point improvement)
Silverberg et al., [22] (2020)	Australia, Bulgaria, Canada, China, Czechia, Germany, Hungary, Japan, South Korea, Latvia, Poland, UK, USA	Phase 3, double-blind, placebo-controlled, parallel-group RCT	Adolescents: ≥12 years (subset not separately reported); total analyzed: 391 patients (155 abrocitinib 200 mg, 158 abrocitinib 100 mg, 78 placebo)	12–17 years (adolescents included), adults also included; mean age 35.1 years (SD 15.1)	Abrocitinib 100 mg or 200 mg orally once daily for 12 weeks	Placebo	12 weeks	IGA response, EASI-75 at week 12
Simpson et al., [23] (2020)	Australia, Canada, Europe, USA	Multicentre, double-blind, randomized phase 3 trial	387 total: adolescents ≥12 years included (exact adolescent count not separately specified)	≥12 years	Abrocitinib 100 mg once daily, Abrocitinib 200 mg once daily, 12 weeks	Placebo	12 weeks	1. Investigator Global Assessment response (0/1 with ≥2-grade improvement from baseline) 2. EASI-75 (≥75% improvement in EASI score from baseline)
Wollenberg et al., [24] (2022)	Multinational	Phase 3 RCTs + Open-label extension (post hoc analysis)	471 (Children: 304; Adolescents: 167)	6–<18 years (Children 6–<12, Adolescents 12–<18)	Dupilumab ± topical corticosteroids, dose not specified, 16-week RCTs + 1-year open-label extension	Placebo ± topical corticosteroids	16 weeks (RCTs) and 1 year (OLE)	SCORAD, objective SCORAD, SCORAD components, SCORAD-50, pruritus, sleep loss

The interventions evaluated included biologic agents-the interleukin (IL)-4/IL-13 receptor antagonist dupilumab [13,14,24] and the IL-13 inhibitor tralokinumab [12], as well as JAK inhibitors, abrocitinib [15,21-23] and upadacitinib [16-20]. Most JAK inhibitor trials and one dupilumab trial [13] allowed concomitant use of TCS, which could act as a potential confounder when interpreting the efficacy outcomes. The comparators were predominantly placebo, often with the option of using background TCS. The primary efficacy outcomes across studies were similar, most commonly including the proportion of patients achieving a 75% or greater improvement in the Eczema Area and Severity Index (EASI-75), an Investigator’s Global Assessment (IGA) score of 0 (clear) or 1 (almost clear) with a ≥2-grade improvement, and meaningful improvements in pruritus as measured by various itch numeric rating scales (NRS).

Efficacy Outcomes

The efficacy outcomes of the included systemic therapies are detailed in Table 2.

**Table 2:** Efficacy and Safety Outcomes of Systemic Therapies AD: Atopic Dermatitis; AE: Adverse Event; CK: Creatine Kinase; CPK: Creatine Phosphokinase; EASI: Eczema Area and Severity Index; EASI-50/75/90: 50%/75%/90% improvement in EASI; ECZTRA: Eczema Tralokinumab Clinical Trial Program; GI: Gastrointestinal; IGA: Investigator’s Global Assessment; LSM: Least Squares Mean; NRS: Numeric Rating Scale; PP-NRS4: Peak Pruritus Numeric Rating Scale (≥4-point improvement); PY: Patient-Years; RCT: Randomized Controlled Trial; SCORAD: Scoring Atopic Dermatitis; SCORAD-50: 50% reduction in SCORAD; TB: Tuberculosis; TEAE: Treatment-Emergent Adverse Event; TCS: Topical Corticosteroids; URTI: Upper Respiratory Tract Infection; vIGA-AD: Validated Investigator’s Global Assessment for Atopic Dermatitis; WP-NRS: Worst Pruritus Numeric Rating Scale; WSI-NRS: Worst Scratch/Itch Numeric Rating Scale

Author (Year)	Intervention	Key Efficacy Outcomes (EASI-50, EASI-75, SCORAD, IGA response, etc.)	Safety Profile (Common Adverse Events)	Serious Adverse Events/Discontinuations
Paller et al., [12] 2025	Tralokinumab (150/300 mg) (ECZTRA 6, adolescents, IGA >1)	- EASI-50: 31.2% / 41.3% vs 10% (placebo) - Itch NRS ≥3-pt: 21.6% / 22.8% vs 8% - ≥1 meaningful response: 36.4% / 52.5% vs 21.1% - Week 52: majority EASI-50 & itch relief; ~40% EASI-90	NR	NR
Ebisawa et al., [13] (2024)	Dupilumab (with TCS, 16 weeks; then open-label to 52 weeks)	- EASI-75 at Week 16: 43.3% vs 18.8% (placebo), P = 0.0304 - Percent change in EASI: LSM difference -39.4%, P = 0.0003 - IGA 0/1 at Week 16: 10.0% vs 9.4% (NS) - Worst daily itch NRS: LSM difference -33.3%, nominal P = 0.0117	Well tolerated; no new safety signals reported	NR
Boguniewicz et al., [14] (2024)	Dupilumab (6 mo–5 yrs, LIBERTY AD PRESCHOOL)	↑ IGA 0/1, ↑ EASI-75, ↓ itch (WSI-NRS)	Consistent with known profile (e.g., conjunctivitis, injection-site reactions, nasopharyngitis)	NR
Paller et al., [15] (2025)	Abrocitinib 200 & 100 mg	EASI-75: 85% / 83%, EASI-90: 62% / 60%, IGA 0/1: 57% / 57% (Week 112)	TEAEs reported; common AEs not specified	Serious TEAEs: 5.47 / 3.45 per 100 PY; Discontinuations: 6.78 / 5.39 per 100 PY
Paller et al., [16] (2024)	Upadacitinib 15 mg & 30 mg once daily	EASI-75 82.7–96.1%, vIGA-AD 0/1 & WP-NRS ≥4-point improvement maintained through 76 weeks	Herpetic infection 1.1–4.0/100 patient-years, CK elevation 7.1–11.6/100 patient-years	No new safety signals; serious AEs not specified
Katoh et al., [17] (2023)	Upadacitinib 15/30 mg + topical corticosteroids	EASI-75/90, vIGA-AD 0/1, ≥4-point WP-NRS maintained through 112 weeks	Acne, nasopharyngitis, herpes zoster	Rare serious AEs (rectal cancer, cerebellar hemorrhage), discontinuations infrequent
Katoh et al., [18] (2022)	Upadacitinib 15–30 mg + TCS	Not reported	Acne (13.2–19.8%), herpes zoster, anemia, neutropenia, CPK elevation (mostly mild/moderate)	Serious adverse events 56–64%; no discontinuations due to AEs; no thromboembolic events, malignancies, GI perforations, TB, or deaths
Reich et al., [19] (2021)	Upadacitinib 15 mg & 30 mg + Topical Corticosteroids	EASI-75: 65% (15 mg), 77% (30 mg); vIGA-AD response: 40% (15 mg), 59% (30 mg) vs placebo 26%/11%	Acne, nasopharyngitis, URTI, oral herpes, elevated CPK, headache, AD	Serious AE: 1–2%; Discontinuation: 1–2%
Guttman-Yassky et al., [20] (2021)	Upadacitinib 15 mg & 30 mg daily (16 weeks)	EASI-75: 60–80%; vIGA-AD response: 39–62%	Acne, URTI, nasopharyngitis, headache, elevated CPK, AD flare	Similar to placebo; no increase in serious AEs or discontinuations
Eichenfield et al., [21] (2021)	Abrocitinib 200 mg & 100 mg + Topical Therapy vs Placebo	IGA 0/1: 46.2% / 41.6% vs 24.5% EASI-75: 72.0% / 68.5% vs 41.5% PP-NRS4: 55.4% / 52.6% vs 29.8%	Nausea: 18.1% / 7.4% (vs not reported in placebo) Overall AEs: 62.8% / 56.8% vs 52.1%	Serious AEs: 1 (1.1%) / 0 vs 2 (2.1%)
Silverberg et al., [22] (2020)	Abrocitinib 200 mg/day & 100 mg/day	IGA response 38.1% / 28.4%, EASI-75 61.0% / 44.5%, EASI-90 37.7% / 23.9%, PP-NRS ≥4 improvement 55.3% / 45.2%	Adverse events 65.8% / 62.7%	Serious AEs 1.3% / 3.2%; platelet decrease/thrombocytopenia in 1.3–3.2%
Simpson et al., [23] (2020)	Abrocitinib 100 mg & 200 mg once daily	IGA response: 24% (100 mg), 44% (200 mg) vs 8% placebo; EASI-75: 40% (100 mg), 63% (200 mg) vs 12% placebo	AEs: 69% (100 mg), 78% (200 mg) vs 57% placebo	Serious AEs: 3% (both doses) vs 4% placebo; no treatment-related deaths
Wollenberg et al., [24] (2022)	Dupilumab ± Topical Corticosteroids	SCORAD ↓ from week 3; SCORAD-50: 91–92% at week 52; pruritus & sleep loss mild/absent >86%	NR	NR

Dupilumab demonstrated significant efficacy in pediatric populations. In Japanese children and adolescents (≥6 months to <18 years), dupilumab with TCS resulted in a significantly higher proportion of patients achieving EASI-75 at week 16 compared to placebo (43.3% vs. 18.8%; p = 0.0304) and a significantly greater least squares mean (LSM) reduction in EASI score (LSM difference -39.4%; p = 0.0003) [13]. Similarly, in children aged six months to five years, dupilumab treatment led to improvements in IGA 0/1, EASI-75, and worst scratch/itch NRS over 16 weeks [14]. A post-hoc analysis of pediatric patients (6-<18 years) showed that dupilumab, with or without TCS, provided rapid and sustained improvement, with 91-92% achieving SCORAD-50 (a 50% improvement in SCORAD) by week 52, and over 86% of patients experiencing mild or absent pruritus and sleep loss [24].

Tralokinumab, another monoclonal antibody, showed efficacy in adolescents who did not achieve clear or almost clear skin by week 16 on the drug. At the primary timepoint, significantly more patients on tralokinumab 150 mg and 300 mg achieved EASI-50 (31.2% and 41.3% vs. 10% placebo) and a ≥3-point improvement in itch NRS (21.6% and 22.8% vs. 8% placebo). By week 52, the majority of these initial non-responders achieved EASI-50 and meaningful itch relief, with approximately 40% achieving EASI-90 [12].

The JAK inhibitors abrocitinib and upadacitinib demonstrated high levels of efficacy, often with rapid onset. In adolescents, abrocitinib at 200 mg and 100 mg doses showed high EASI-75 response rates at week 12 (63% and 40% vs. 12% placebo in one trial [23]; 72.0% and 68.5% vs. 41.5% placebo in another [21]). Long-term data through 112 weeks showed maintained efficacy, with EASI-75 rates of 85% and 83% for the 200 mg and 100 mg doses, respectively, and 57% of patients in both groups achieving IGA 0/1 [15].

Upadacitinib efficacy was consistently high across trials. In adolescents, doses of 15 mg and 30 mg once daily, often with TCS, resulted in EASI-75 rates ranging from 60% to over 96% at various timepoints [16,19,20]. Reich et al. [19] reported EASI-75 rates of 65% (15 mg) and 77% (30 mg) vs. 26% (placebo) at week 16. Long-term analyses confirmed that this efficacy was maintained through 76 [16] and 112 weeks [17]. Furthermore, a significant proportion of patients achieved higher thresholds of response, such as EASI-90, and sustained improvements in pruritus [16,17].

Safety and Tolerability

The safety profiles of the interventions were generally consistent with their known pharmacological class effects.

Dupilumab was reported to be well-tolerated across studies. The most common adverse events (AEs) included conjunctivitis, injection-site reactions, and nasopharyngitis [14]. Ebisawa et al. [13] reported no new safety signals in their Japanese pediatric population.

Tralokinumab's safety profile was not detailed (NR) in the included abstract [12]. The most frequently reported AEs for abrocitinib included nausea, which was dose-dependent (18.1% with 200 mg vs. 7.4% with 100 mg vs. not reported in placebo in one trial [21]), and overall AEs were more common than with placebo [22,23]. Serious AEs were generally low (1.3-3.2%) and comparable to placebo [22,23], though one study noted platelet decrease/thrombocytopenia in 1.3-3.2% of patients [22]. Long-term safety data over a median exposure of ~2.6-2.7 years reported serious treatment-emergent AE (TEAEs) at rates of 5.47 and 3.45 per 100 patient-years for the 200 mg and 100 mg doses, respectively [15].

Upadacitinib was associated with AEs such as acne, nasopharyngitis, upper respiratory tract infections, herpes simplex and zoster infections, elevations in creatine phosphokinase (CPK), and headache [16-20]. These events were mostly mild-to-moderate in severity. Herpetic infection rates were reported between 1.1-4.0 per 100 patient-years and CPK elevation between 7.1-11.6 per 100 patient-years [16]. While serious AEs were generally uncommon (e.g., 1-2% in [19]), one interim analysis reported a high percentage of serious AEs (56-64%); however, these were not attributed to the drug, and there were no discontinuations due to AEs, nor any thromboembolic events, malignancies, gastrointestinal perforations, tuberculosis, or deaths [18]. Rare serious AEs, such as rectal cancer and cerebellar hemorrhage, were reported in one patient each but were considered unrelated to the study drug [17]. Discontinuation rates due to AEs were low across upadacitinib studies [19,20].

Risk of Bias Results

The methodological quality of the included studies was generally high, as assessed by the Cochrane ROB 2 tool. The majority of studies were judged to have a low risk of bias across all domains, including the randomization process, deviations from intended interventions, missing outcome data, measurement of the outcome, and selection of the reported result [12-21,23,24]. One study by Silverberg et al. [22] was rated as having "some concerns" overall, which originated from the measurement of the outcome domain, potentially due to the subjective nature of the primary endpoints and the possibility of unblinding. Consequently, the body of evidence synthesized in this review demonstrates a consistently low risk of bias, with concerns isolated to a single study (Table 3)

**Table 3:** Cochrane ROB 2 Risk of Bias Assessment Results

Author (Year)	Randomization Process	Deviations from Intended Interventions	Missing Outcome Data	Measurement of the Outcome	Selection of the Reported Result	Overall RoB
Paller et al., [12] 2025	Low	Low	Low	Low	Low	Low
Ebisawa et al., [13] (2024)	Low	Low	Low	Low	Low	Low
Boguniewicz et al., [14] (2024)	Low	Low	Low	Low	Low	Low
Paller et al., [15] (2025)	Low	Low	Low	Low	Low	Low
Paller et al., [16] (2024)	Low	Low	Low	Low	Low	Low
Katoh et al., [17] (2023)	Low	Low	Low	Low	Low	Low
Katoh et al., [18] (2022)	Low	Low	Low	Low	Low	Low
Reich et al., [19] (2021)	Low	Low	Low	Low	Low	Low
Guttman-Yassky et al., [20] (2021)	Low	Low	Low	Low	Low	Low
Eichenfield et al., [21] (2021)	Low	Low	Low	Low	Low	Low
Silverberg et al., [22] (2020)	Low	Low	Low	Some concerns	Low	Some concerns
Simpson et al., [23] (2020)	Low	Low	Low	Low	Low	Low
Wollenberg et al., [24] (2022)	Low	Low	Low	Low	Low	Low

Discussion

This systematic review provides a comprehensive synthesis of the current evidence from 13 clinical trials evaluating the efficacy and safety of advanced systemic therapies - specifically the biologics dupilumab and tralokinumab, and the JAK inhibitors abrocitinib and upadacitinib - for the management of moderate-to-severe AD in children and adolescents. The collective findings from these studies robustly demonstrate that these targeted therapies represent a paradigm shift in pediatric AD management, offering significant and often rapid improvements in disease signs, symptoms, and quality of life for a population with historically limited and unsatisfactory treatment options. The high efficacy observed across all drug classes is particularly noteworthy. Dupilumab consistently showed significant benefits over placebo, with EASI-75 response rates of 43.3% at week 16 in a Japanese pediatric cohort [13] and remarkable long-term control, as evidenced by SCORAD-50 rates exceeding 90% at one year in a multinational analysis [24]. This aligns with and extends the established efficacy profile of dupilumab from adult studies and earlier pediatric trials. For instance, the landmark phase 3 trials in adolescents (LIBERTY AD ADOL) previously established its efficacy and safety, leading to its initial approval in this age group [25]. Our review consolidates these findings and confirms its effectiveness across a broader pediatric spectrum, down to children as young as six months [14].

The efficacy of the IL-13 inhibitor tralokinumab, while demonstrated in a specific subgroup of adolescent initial non-responders, is a significant addition to the treatment landscape [12]. The fact that a substantial proportion of these difficult-to-treat patients went on to achieve meaningful clinical responses, including EASI-90, by week 52 suggests a valuable role for this biologic, potentially offering an alternative mechanism of action for patients who may not fully respond to IL-4/IL-13 receptor blockade. This finding warrants further investigation in head-to-head trials against dupilumab to better delineate its position in the treatment algorithm.

Perhaps the most striking efficacy signals came from the JAK inhibitors, abrocitinib and upadacitinib. These oral agents demonstrated rapid and high-magnitude responses, with EASI-75 rates frequently exceeding 70% and even reaching over 90% for upadacitinib 30 mg in some studies by week 16 [16,19,20]. The speed of action, particularly on pruritus, is a defining characteristic of this drug class. The rapid and significant reduction in itch NRS scores observed across JAK inhibitor trials [16,17,21,22] addresses one of the most debilitating symptoms of AD and is a crucial advantage for severely affected patients. This aligns with the known mechanism of JAK inhibitors, which broadly and quickly interrupt the signaling of multiple cytokines involved in the pathogenesis of AD and the pruritus pathway. The high efficacy seen in our reviewed studies is consistent with data from adult populations. For example, the consistent EASI-75 and IGA 0/1 responses for upadacitinib mirror those reported in the Measure Up 1 & 2 and AD Up trials in adults [20,26], while the abrocitinib efficacy data corroborate findings from the JADE MONO-1 and MONO-2 trials [25,27]. Our review confirms that this high level of efficacy translates effectively to the adolescent population and can be maintained over extended treatment periods of up to 112 weeks [15,17].

However, this superior efficacy must be carefully balanced against the distinct safety profiles of each drug class, a central tenet of any discussion on modern AD therapeutics. The safety data compiled in this review reaffirm the well-characterized profiles of these agents. Dupilumab maintains a favorable safety profile with mostly mild-to-moderate AEs such as conjunctivitis and injection-site reactions [13,14,24]. The absence of new safety signals in these pediatric studies is reassuring and reinforces its position as a first-line systemic biologic with a well-established risk-benefit ratio, a conclusion supported by long-term real-world studies in both adults and children [28-30].

The safety profile of the JAK inhibitors is more complex and requires nuanced interpretation. The higher incidence of certain AEs, such as acne, nausea (for abrocitinib), and herpes zoster infections, is consistent with the pharmacodynamics of JAK inhibition [16,18,19,22]. These are largely manageable in a clinical setting. However, the findings of laboratory abnormalities, including elevated CPK and, notably, dose-dependent platelet decrease with abrocitinib [22], underscore the necessity for routine monitoring, as recommended by regulatory authorities. The occurrence of serious AEs, though generally low and comparable to placebo in the controlled periods of these trials [19,22,23], and the report of rare but serious events such as rectal cancer and cerebellar hemorrhage (deemed unrelated) [17,18] highlight the critical importance of ongoing pharmacovigilance. The recent boxed warnings for JAK inhibitors regarding thrombosis, malignancy, major adverse cardiovascular events, and mortality, based largely on data from studies in rheumatoid arthritis patients with cardiovascular risk factors, must be contextually applied. The patients in the included pediatric AD trials were generally young and without such comorbidities, and no such signals were detected in the study periods [18]. Nevertheless, this mandates a cautious approach, careful patient selection, and adherence to risk mitigation strategies, especially for long-term use. This safety discourse is reflected in current international guidelines, which often position JAK inhibitors after biologics or for patients who require a rapid response, reflecting the ongoing evaluation of their risk-benefit profile in younger populations [31].

When comparing the two drug classes, the choice between a biologic and a JAK inhibitor often involves a trade-off between route of administration, speed of response, relapse rate, and safety considerations. Biologics offer a reassuring long-term safety profile and quarterly or monthly dosing after loading, which may improve adherence for some. JAK inhibitors provide the convenience of oral administration and often a faster and more potent initial response, particularly for itch, but require daily dosing and more intensive laboratory monitoring. There are no head-to-head trials comparing these agents in pediatric populations, making direct comparisons from this review impossible. However, network meta-analyses in adults have suggested that high-dose JAK inhibitors may have a comparative efficacy advantage over dupilumab in the short term [29], a finding that, if extrapolated with caution, may inform clinical decision-making in severe adolescent cases where rapid disease control is paramount.

The generalizability of our findings is strengthened by the multinational nature of many included trials [12,16,19,20,24] and data from specific populations, such as Japanese patients [13,17,18]. The consistent results across diverse geographic and ethnic backgrounds suggest that the efficacy and safety of these therapies are broadly applicable. Furthermore, the inclusion of long-term extension studies [12,15-17,24] provides invaluable evidence regarding the durability of response and the maintenance of safety over time, which is critical for a chronic condition such as AD that requires prolonged management.

The strength of this systematic review lies in its comprehensive and focused synthesis of high-quality RCTs specifically conducted in pediatric and adolescent populations - a group often underrepresented in dermatological research. By integrating efficacy, safety, and long-term outcomes across multiple systemic therapies, this review offers clinically relevant insights that support evidence-based treatment decisions and highlight existing research gaps for future studies.

Limitations

This systematic review has several limitations. Firstly, the included studies, while high quality, were primarily industry-sponsored clinical trials with strict inclusion and exclusion criteria. Consequently, the study populations may not be fully representative of the "real-world" pediatric AD population, which often includes patients with more comorbidities, complex psychosocial backgrounds, and concurrent medications. Secondly, the absence of head-to-head trials between the different systemic therapies limits our ability to make definitive comparative conclusions about which drug is most effective or safest for specific patient subtypes. Thirdly, the follow-up duration, while extending to two years in some studies, is still insufficient to fully capture very long-term risks, particularly the potential for rare AEs or the impact of decades of JAK inhibition in a developing child. Finally, the reliance on published data and abstracts means that some granular safety and efficacy data were not available for a more detailed analysis.

## Conclusions

Dupilumab, tralokinumab, abrocitinib, and upadacitinib are highly effective therapeutic options for children and adolescents with moderate-to-severe AD, a condition that significantly impairs quality of life. Dupilumab offers a strong efficacy profile with a well-established and favorable safety record. The JAK inhibitors, particularly upadacitinib, demonstrate superior efficacy and rapidity of response, especially for severe itch, but their use requires careful consideration of safety profiles and monitoring requirements. The choice of agent should be individualized, taking into account disease severity, the urgency of symptom control, patient and caregiver preferences regarding route of administration, comorbidity profile, access to monitoring resources, and treatment cost, which can influence feasibility and adherence. Future research should prioritize head-to-head comparative trials, long-term real-world safety studies, and investigations into predictive biomarkers to guide personalized therapy selection in pediatric AD.

## Appendices

**Table 4:** Full Search Strings for All Databases

Database	Search String
PubMed/MEDLINE	("Atopic Dermatitis"[Mesh] OR "eczema"[tiab]) AND ("children"[Mesh] OR "adolescents"[Mesh] OR "pediatric"[tiab] OR "child*"[tiab] OR "adolescent*"[tiab]) AND ("dupilumab"[tiab] OR "tralokinumab"[tiab] OR "abrocitinib"[tiab] OR "upadacitinib"[tiab] OR "JAK inhibitor*"[tiab] OR "biologic*"[tiab] OR "systemic therapy"[tiab]) AND ("Randomized Controlled Trial"[pt] OR "RCT"[tiab]) AND ("2020/01/01"[Date - Publication] : "2025/12/31"[Date - Publication])
Scopus	TITLE-ABS-KEY("atopic dermatitis" OR eczema) AND TITLE-ABS-KEY(child* OR adolescent* OR pediatric) AND TITLE-ABS-KEY(dupilumab OR tralokinumab OR abrocitinib OR upadacitinib OR "JAK inhibitor*" OR biologic* OR "systemic therapy") AND (LIMIT-TO(DOCTYPE,"ar")) AND (LIMIT-TO(PUBYEAR,2020-2025)) AND (LIMIT-TO(SRCTYPE,"j"))
Web of Science	TS=("atopic dermatitis" OR eczema) AND TS=(child* OR adolescent* OR pediatric) AND TS=(dupilumab OR tralokinumab OR abrocitinib OR upadacitinib OR "JAK inhibitor*" OR biologic* OR "systemic therapy") AND DOCUMENT TYPES=(Article) AND PUBLICATION YEARS=2020-2025
ClinicalTrials.gov	Condition/Disease: "Atopic Dermatitis" AND Other Terms: ("dupilumab" OR "tralokinumab" OR "abrocitinib" OR "upadacitinib" OR "JAK inhibitor*" OR biologic* OR "systemic therapy") AND Age: "Child" OR "Adolescent" AND Study Type: "Interventional" AND Study Results: "All Studies"
